# The Importance of Lifting Height and Load Mass for Muscular Workload during Supermarket Stocking: Cross-Sectional Field Study

**DOI:** 10.3390/ijerph19053030

**Published:** 2022-03-04

**Authors:** Sebastian Venge Skovlund, Rúni Bláfoss, Sebastian Skals, Markus Due Jakobsen, Lars Louis Andersen

**Affiliations:** 1 National Research Centre for the Working Environment, DK-2100 Copenhagen, Denmark; rub@nfa.dk (R.B.); sls@nfa.dk (S.S.); mdj@nfa.dk (M.D.J.); lla@nfa.dk (L.L.A.); 2 Research Unit for Muscle Physiology and Biomechanics, Department of Sports Science and Clinical Biomechanics, University of Southern Denmark, DK-5230 Odense, Denmark; 3 Sport Sciences-Performance and Technology, Department of Health Science and Technology, Aalborg University, DK-9220 Aalborg, Denmark

**Keywords:** musculoskeletal diseases, manual material handling, grocery stores, lifting, EMG, retail industry

## Abstract

High physical work demands increase the risk of musculoskeletal disorders and sickness absence. Supermarket work involves a high amount of manual material handling. Identifying specific ergonomic risk factors is an important part of occupational health and safety efforts in the supermarket sector. In this cross-sectional field study among 64 supermarket workers, we used electromyography during the workday to determine the influence of lifting height and load mass on muscular workload of the low-back and neck/shoulder muscles during un-restricted manual material handling (grocery stocking). We found a significant effect of load mass, i.e., higher loads associated with higher muscular workload in the low-back and neck/shoulder muscles. We demonstrated a significant interaction between start and end position, i.e., lifts performed from ‘Low’ start positions to ‘High’ end positions demonstrated the highest low-back muscular workload, whereas ‘High’ positions were associated with increased neck/shoulder workload. In conclusion, lifting higher loads and lifting goods from low to high positions (low-back) and at high positions (neck/shoulder) are associated with higher muscular workload. These results can be used to guide highly warranted preventive initiatives to reduce the physical workload during supermarket work.

## 1. Introduction

Ergonomic risk factors in the working environment and overall high physical demands at work constitute important risk factors for the development and aggravation of musculoskeletal disorders (MSD) [1,2,3], sickness absence [4,5], and disability pension [6,7]. High physical workload, e.g., high work exposure to repetitive arm movement, high force exertion, kneeling, squatting, or lifting, remains highly prevalent globally [8,9] and, thereby, imposes a substantial global burden on individuals, workplaces, and socio-economics [10,11]. Identifying and handling ergonomic risk factors at the workplace could potentially reduce this burden and, therefore, has become a major political priority [12].

Physically demanding work tasks, such as manual material handling (MMH), are highly common during supermarket stocking, which may partly explain the high prevalence of MSD among supermarket workers, especially in the low-back and neck/shoulder [13,14,15,16,17,18,19,20]. Hitherto, traditional ergonomic approaches focusing on handling technique have proven largely ineffective for the prevention and rehabilitation of MSD [21,22]. 

Instead, it may be worthwhile to focus on workplace factors that dictate the physical demands needed to fulfill a given MMH task. For instance, MMH at high heights often requires working with arms above shoulder level, which is a well-established risk factor for MSD in the neck and shoulders [3,23]. Likewise, low handling heights during supermarket stocking increase low-back loading and require high degrees of trunk flexion [24,25], which has been shown to increase the risk of low-back pain [26,27]. Finally, handling goods in awkward work postures has also been associated with an increased risk of low-back pain [2,28]. Thus, the high physical workload, i.e., muscular strain, associated with high exposure to these ergonomic work postures entails an increased risk of MSD. Therefore, identifying specific workplace factors associated with high physical workload may be an essential step in workplace-based prevention of MSD, sickness absence, and disability pension.

The assessment of physical workload during MMH activities has predominantly relied on self-reports or observational methods [29], which also pertains to the assessment of physical workload during supermarket work [13,17,18,20]. Although these methodological approaches have merit under certain circumstances, assessing physical workload by means of self-reports or observational methods generally demonstrate lower validity compared to technical measurements [30,31]. Therefore, more research is needed employing technical measurements to assess the physical workload.

Multiple biomechanical studies have assessed the influence of certain MMH factors in terms of physical workload, e.g., lifting height [32,33,34,35,36,37,38] and/or load mass [32,33,34,35,37,38,39]. However, many of these studies were conducted in laboratories under standardized conditions, which does not capture the complexity as well as intra- and inter-subject variability of un-restricted or real-life work practices. Exemplifying the pitfalls of laboratory-based investigations of real-life work exposures, Moriguchi and colleagues investigated the agreement in work postures, e.g., neck flexion/extension and upper arm elevation, recorded during simulated work tasks in a laboratory and the same work tasks performed in an un-restricted field environment [40]. The authors found that two simpler work tasks presented similar exposure in both conditions, whereas differences between conditions were reported for a more complex task (relay replacement), indicating insufficient reproduction of the field exposure. As another example, Faber and colleagues demonstrated six percent lower peak low-back (L5/S1 joint) moments during a typical laboratory-based lifting task compared to a more realistic task involving carrying the same load for a short distance [41]. These examples of troublesome extrapolation of real-life field exposures based on laboratory-based studies underline the importance of conducting risk assessment studies in as natural an environment as possible. As examples hereof, our lab has previously conducted comprehensive field investigations of muscular workload by use of portable electromyography (EMG) and accelerometers during full workdays of construction workers and nurses [42,43], but studies such as these are lacking in the supermarket sector. Thus, more field studies are needed using technical measurements to determine the importance of certain lifting factors for the physical workload during un-restricted supermarket work, in order to develop effective preventive initiatives for workplaces and work environment professionals aiming to reduce the burden of high physical workload and MSD.

In this field study, we combined surface electromyography (sEMG) and video recordings to estimate the influence of lifting height and load mass on the muscular workload of the low-back and neck/shoulder muscles during un-restricted MMH activities in supermarkets. We hypothesized that these lifting factors, especially in combination, would exert high influence on the muscular workload. Identifying factors at the supermarkets associated with high physical workload during un-restricted supermarket work could be valuable in terms of guiding preventive initiatives, for instance concerning re-design of store layouts and re-organization of the work. Thereby, these results could be important for workplace-based prevention of MSD, sickness absence, and disability pension in the supermarket sector.

## 2. Materials and Methods

### 2.1. Study Design and Setting

**Figure 1:**
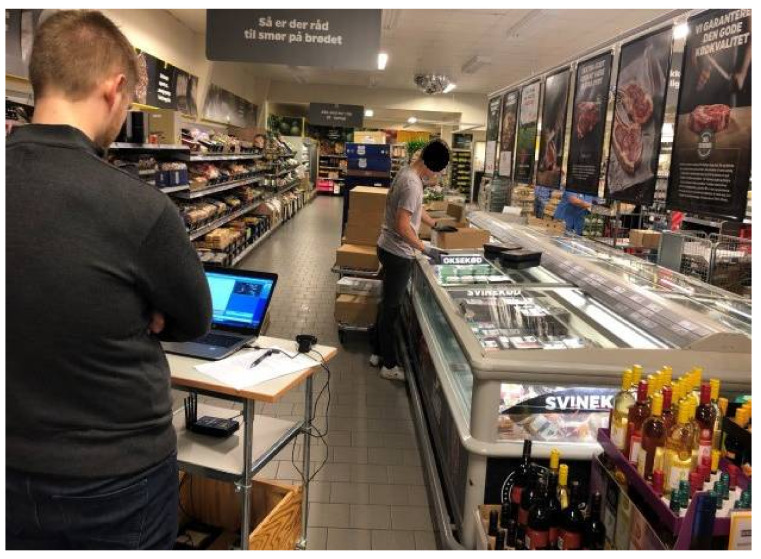
Measurements of muscular workload using sEMG and synchronous video recording.

In accordance with the STROBE (Strengthening the Reporting of Observational Studies in Epidemiology) reporting guidelines [44], this cross-sectional field study reports the estimates of muscular workload in relation to lifting height and load mass during grocery stocking among Danish supermarket workers (*n* = 75) from five different supermarket chains [45]. Research collaborators from The Danish Chamber of Commerce contacted and recruited representatives from interested supermarket chains, who were responsible for the recruitment of volunteering supermarket stores. Data were collected between December 2018 and July 2019. All experimental procedures were performed at the supermarkets and lasted approximately three hours for each participant, starting with informal consent and instrumentation of the sEMG equipment on the participant, followed by normalization of sEMG (more details below), recording of sEMG and video during work tasks in the store (see Figure 1), normalization of sEMG after recording in the store, and ultimately dismounting the sEMG equipment while debriefing.

### 2.2. Participants

Potential participants received written information about the research project prior to enrolment. In the present study, we included adult supermarket workers (≥18 years) that had been working roughly full-time (≥30 h per week) for a minimum of six months, and excluded candidates with severe cardiovascular disease, ambulatory systolic/diastolic blood pressure ≥160/100 mmHg, and pregnancy [43]. All participants (*n* = 75) were asked to reply to an electronic questionnaire regarding their work environment, lifestyle, and health, and the entire questionnaire and replies have been published previously [45]. Response rate for completing the entire questionnaire was 89% (*n* = 67). Complete data was not available for all participants, which explains why the exact number of participants for each analysis varies.

### 2.3. Ethical Approval

The Danish National Committee on Biomedical Research Ethics (The local ethical committee of Frederiksberg and Copenhagen; H-3-2010-062) approved the study. Complying with the Helsinki Declaration, all participants received both written and oral information about the study, potential risks related to the measurements, and their rights before giving oral and written informed consent. The National Research Centre for the Working Environment has a collective agreement with the Danish Data Protection Agency about data handling procedures compliant with the General Data Protection Regulation. Thereby, the in-house responsible person approved the study before initiating data collection. Data were handled and analyzed anonymously from a secure server at the National Research Centre for the Working Environment.

### 2.4. Operationalization of Lifting Height and Load Mass

During data collection, all participants performed approximately 1½–2 h of un-restricted MMH (grocery stocking), while the principal investigators (SVS and RB) carried out the technical measurements and simultaneously video recorded the work activities (see Figure 1) [45]. During data analysis, we used the video recordings to categorize the lifting tasks by lifting height and load mass (see below).

### 2.5. Lifting Height

By visually inspecting the video recordings, we categorized the start and end positions of the lifting tasks into three different heights: low, medium, and high.

Lifting heights were based on average-sized body segments. Thus, ‘Low’ height was defined as any lift initiated or terminated below an average-sized worker’s hip, whereas ‘High’ was defined as lifts of goods handled with the hands at or above shoulder height, with ‘Mod’/moderate heights representing lifts carried out between these two extremes, i.e., from the hip to the shoulder level.

### 2.6. Load Mass

To categorize the load mass of the handled goods, we weighed numerous of the most common goods within different product lines as well as relied on product declarations, e.g., assuming 1 L of milk weighing around 1 kg. If neither option was feasible, the assignment of the load handled was based on our best estimate. If our uncertainty was too high, the lifting sequence was not assigned a load mass. We noted the known/exact mass whenever possible, e.g., handling of two one-liter milks weighing two kg, and assigned load mass intervals when the exact mass was not known. We used the following load mass intervals: 0–1 kg, ≥1–5 kg, ≥5–10 kg, ≥10–15 kg, and ≥15 kg.

### 2.7. Experimental Design

We assessed muscular workload (based on myoelectric activity) using an experimental protocol previously applied and described by our lab [43,46]. Briefly, we combined sEMG measurements with simultaneous video recordings of the stocking activities in the supermarkets [45]. We measured muscular workload of the m. erector spinae longissimus, m. iliocostalis, and m. trapezius descendens, since these body regions (the low-back and neck/shoulders) are highly susceptible to MSD in general [47] and among supermarket workers [13,14,15,16,17,18,19,20].

**Figure 2:**
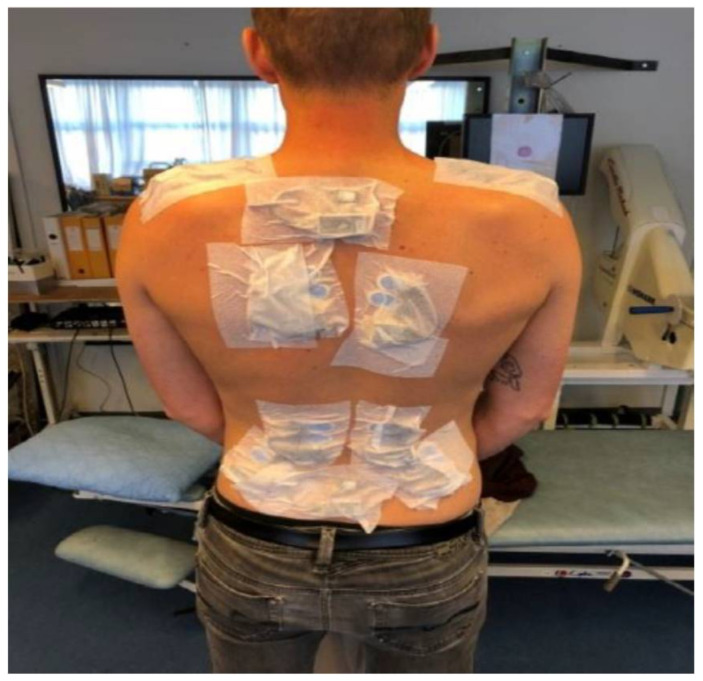
Example of placement of sEMG electrodes fixated with Fixomull.

Bipolar sEMG were recorded wirelessly (TeleMyo DTS Telemetry, Noraxon, AZ, USA) at a sampling rate of 1500 Hz and a bandwidth of 10–500 Hz, with the amplifier having a 16-bit A/D converter and a common mode rejection ratio >100 dB. Before instrumentation of electrodes, the skin was cleaned and prepared using scrubbing gel (Acqua gel, Meditec, Parma, Italy) to reduce skin impedance. Afterwards, electrodes (Blue Sensor N-00-S, Ambu A/S, Ballerup, Denmark) were placed bilaterally on the m. trapezius descendens, m. erector spinae longissimus, and m. iliocostalis with an inter-electrode distance of two centimeters [48]. Electrodes and cables were fixated to the skin using stretch tape (Fixomull), see Figure 2.

### 2.8. Maximal Voluntary Isometric Contractions

After attaching the electrodes and before initiating the measurements, sEMG normalization procedures were performed for the m. erector spinae (lying in the Biering-Sørensen position [49,50]) and upper trapezius muscles [25,43], which consisted of maximal voluntary isometric contractions (MVIC) [51,52]. The latter was performed standing in an upright position with arms held at 90 degrees abduction [46]. Participants performed three MVIC trials separated by one minute rest. The test leader informed the participants to progressively produce more force before reaching their maximum within 2–3 s, and the MVIC was terminated when the test leader informed the participant to stop, or the participant reached exhaustion. During MVICs, the test leader verbally encouraged the participants. After all of the MVIC trials, participants rated their effort on a 0–10 Borg’s rating scale. We repeated this procedure after performing the measurements with the highest recorded muscle activity chosen as the reference value for the sEMG normalization.

During data processing, all raw sEMG signals were digitally filtered through a Butterworth fourth-order high-pass filter (10 Hz cut-off frequency), and full-wave rectified and smoothed using a root-mean-square (RMS) filter with a moving window of 500 ms. In addition, all trials were visually inspected for non-physiological signal artefacts, e.g., spikes, gaps, or low signal-to-noise ratio. For each individual muscle and trial, the 95th percentile of the smoothed RMS signal was normalized (nRMS) to the maximal moving RMS (500-ms time constant) EMG amplitude obtained during the MVICs [53]. The nRMS values of the two bilateral erector spinae muscles (m. longissimus and m. iliocostalis) were merged, resulting in a summed muscular workload for the low-back. Likewise, we merged the nRMS values of the bilateral upper trapezius muscles (m. trapezius descendens) providing summed muscular workload for the neck/shoulder region.

### 2.9. Statistical Analyses

Data were analyzed using linear mixed models with repeated measures (Proc Mixed, SAS v9.4, SAS Institute, Cary, NC, USA). Muscular workload (normalized EMG) was the primary outcome measure. Estimates are reported as least-square means (LSM) with 95% confidence intervals of the 95th percentile rank of nRMS. Alpha levels below 0.05 were considered statistically significant.

The predictive variables were lifting start and end position (and their interaction), and load mass. The predictive variables were mutually controlled for each other. Additionally, all analyses were controlled for participant age (years, continuous variable) and sex (‘male’ or ‘female’, categorical variable). All analyses were stratified for muscle, i.e., low-back muscles and trapezius muscles were analyzed separately. When the exact load mass was known, we categorized the value to fit with the categorical values.

## 3. Results

Table 1 presents participant characteristics. Complete data on lifting height and load mass were available for 64 participants of which 56 completed the questionnaire. The 64 participants were on average 31 years old, 61% were men, and they generally rated themselves as healthy.

### Muscular Workload

The muscular workload of the low-back and neck/shoulders are reported in Table 2, Table 3, Table 4 and Table 5. In addition, Supplementary Tables S1–S4 report differences in least-square means between conditions and their *p*-values.

Overall, we observed a significant effect of load mass and start and end position of the lifts for both the low-back and neck/shoulder muscles (*p* < 0.001). Thus, increments in load mass were generally associated with higher muscular workload of the low-back and neck/shoulders (Table 2). We found significant differences in low-back muscular workload between all load mass intervals, except between intervals 0–1 and ≥1–5 kg. Similarly, all load mass intervals differed significantly in terms of neck/shoulder muscular workload, expect for the ≥5–10 kg and ≥10–15 kg intervals.

Albeit the differences were generally minor compared with the differences between the load mass intervals, there were significant differences in low-back and neck/shoulder muscular workload between all lifting start and end positions, except between the ‘Low’ and ‘Mod’ end positions with respect to low-back muscular workload (Table 3). A ‘High’ start and/or end position demonstrated the highest muscular workload of the neck/shoulders, whereas differences between lifting heights were less pronounced and generally more modest for the low-back muscles. Still, the ‘Low’ start position was associated with the highest low-back muscular workload (27% nEMG, 95% CI: 25–29% nEMG). Importantly, the interaction between start and end position for muscular workload was significant (Table 4 and Table 5), i.e., different combinations of start and end positions influenced the workload differently. Across load mass intervals, lifts performed from ‘Low’ to ‘High’ positions were generally associated with the highest low-back muscular workload, e.g., 58% nEMG (95% CI: 40–76% nEMG) and 46% nEMG (95% CI: 39–52% nEMG) for load mass intervals >10–15 kg and >15 kg, respectively. Lifts performed at ‘High’ start or end positions generally associated with higher neck/shoulder muscular workloads compared to lifts that did not involve ‘High’ start or end positions.

## 4. Discussion

This study used sEMG field measurements to assess the importance of load mass and lifting height for the peak muscular workload of the low-back and neck/shoulder muscles among supermarket workers performing un-restricted stocking activities. Both load mass and start and end position during a given lift influenced the muscular workload. More specifically, lifts performed from ‘Low’ to ‘High’ were associated with a particularly high low-back muscular workload, whereas especially ‘High’ start and end positions demonstrated high workload of the neck/shoulder muscles. These results can guide preventive initiatives to reduce the physical workload during supermarket work.

These field measurements underscore that both load mass and lifting height influence peak muscular workload of the low-back and neck/shoulders. Higher load mass was consistently associated with higher muscular workload for both muscle groups. The positive association between load mass and peak muscular workload during un-restricted stocking supports the results of numerous previous laboratory and field studies reporting paralleled increases in load mass and workload of the lower back, knees, and shoulders [24,25,33,35,36,38,39,54]. Using state-of-the-art musculoskeletal models, Skals and colleagues recently demonstrated a clear positive linear relationship between load mass (five kg increments from 5 to 25 kg) and the peak joint reaction forces of the knee and shoulders, as well as the peak compression (L5/S1) and anteroposterior shear forces of the lumbar spine [38]. Likewise, Plamondon et al. reported significantly different peak lumbar spine moments (L5/S1) between lifting objects with load masses of 15 and 23 kg, respectively [54]. Contradictory, Silvetti et al. did not demonstrate differences in peak EMG of the m. deltoideus anterior or m. erector spinae longissimus between 6 and 8 kg loads, likely due to the relatively small difference between loads and low statistical power resulting from the inclusion of only five participating supermarket workers [37]. However, all of these studies included smaller samples than the present study (*n* from five to 30) and the lifts were performed under standardized conditions, which may have inhibited the subjects from handling the goods as they normally would. Hence, these studies may not have captured the natural intra- and inter-individual variation in lifting technique during real-life MMH, hereby compromising the studies’ external validity [24,25,55]. Nonetheless, the current field study among a large sample of supermarket workers clearly indicated that real-life stocking of supermarket goods of increased load masses was associated with an increased muscular workload in both the low-back and neck/shoulder muscles.

In line with multiple previous reports [24,25,33,35,36,37,38,54], our field study also underpins lifting height as an important lifting factor influencing peak muscular workload. Specifically, our data indicated that ‘High’ lifting start and end positions were associated with particularly increased neck/shoulder muscular workload, e.g., lifts from either ‘Low’ or ‘Mod’ start positions to ‘High’ end positions. Previous studies assessing outcomes such as glenohumeral joint reaction forces [24,38] and peak EMG activity of the m. deltoideus [36,37] and m. trapezius descendens [25] have reported similar findings, e.g., higher neck/shoulder workloads at high lifting heights.

Differences between lifting heights considered in isolation were generally less pronounced in the present study in terms of low-back muscular workload compared to the clearer influence of load mass. Still, the ‘Low’ lifting start position demonstrated the highest low-back peak muscular workload of all lifting height conditions, while no significant difference existed between the ‘Low’ and ‘Mod’ lifting end positions, and only marginal difference existed between the ‘Low’ and ‘High’ lifting end positions. However, the interaction analyses demonstrated that especially lifts performed from ‘Low’ to ‘High’ positions were associated with high low-back muscular workload. Previous studies have demonstrated increased low-back peak workloads (forces/moments) at lower lifting heights compared to higher lifting positions [35,38,54]. However, other studies carried out in a supermarket context have also reported surprisingly high peak EMG activity of the low-back when lifts were performed at or to high lifting heights [25,37]. One explanation for this can be that the workers chose to accelerate the goods from the starting position using their lower back to alleviate their shoulders at high end positions, which may have resulted in a brief moment of high muscular activity [38]. Furthermore, long reaching distances when placing goods at high shelves could also result in high m. erector spinae activity to counteract the increased moment at the lower back. Thus, an important take-home-message from our field study is that ‘High’ lifting heights are associated with both a high neck/shoulder workload and low-back workload, which was also observed in a recent field study in the supermarket sector [25].

Importantly, some studies [36,54], but not all [37,54], have previously reported significant interactions between lifting load and height. Poitras and colleagues found a significant interaction between lifting load and height [36], whereas Silvetti found no such interaction [37]. Plamondon observed an interaction between lifting load and height when the peak forces were obtained during the lifting phase, but not during the deposit phase [54]. Thus, conflicting evidence exist about the interaction between lifting load and height in terms of workload, although most studies observed an interaction.

### 4.1. Practical Applications

Reducing the physical workload associated with supermarket work seems warranted for several reasons previously elaborated. First, high physical workload is associated with MSD across occupations [1,2,3]. Secondly, supermarket work is in general physically demanding [20,24,25,56], and a high prevalence of MSD is often reported among supermarket workers [13,14,15,16,17,18,19,20]. Thus, it is reasonable to speculate that high physical work demands represent one contributing factor to the high prevalence of MSD among supermarket workers. Reducing the physical workload during supermarket work could therefore be one viable strategy to reduce the overall burden of MSD in this group of workers.

Both our field study and other studies conducted under more standardized conditions concordantly underpin lifting height and load mass as important lifting factors determining the physical demands of a given lift. It should be kept in mind that studies (the present study included) investigating physical workload only provide a snapshot of the workers’ working life. The workers are exposed to these lifts during a large part of the working day, several days per week, and for many years. Our lab previously observed a positive exposure-response association between lifting load and low-back pain intensity among supermarket workers [56], indicating a negative short-term effect of accumulative occupational lifting on low-back pain. This load accumulation during the working day and working life underscores the necessity for conducting initiatives to reduce the physical workload in the short- and long-term.

Reducing the load mass of the heaviest parcels could be a good place to start. Skals and colleagues have previously reported load masses of some of the most common goods in a Danish supermarket chain [24,25]. Bananas and milk were by far the heaviest goods included in this study, averaging 20.2 kg and 17.3 kg, respectively. If supermarket chains collectively demanded lowered parcel masses from the suppliers, this could help reduce the peak workload during supermarket stocking. Another solution could be to prioritize stocking fewer goods at a time, e.g., stock one milk at a time instead of lifting the whole box of milk on the shelf. Neither of these possible solutions would, however, reduce the total accumulated workload as the supermarkets’ product range would still have to match customer requirements to remain competitive. This is unfortunate as both peak and cumulative loading have been prospectively associated with MSD [56,57,58]. Recent evidence from the supermarket sector suggests a high potential of workload management on both a daily and weekly basis in terms of low-back pain intensity [56]. Hence, proper organization and distribution of the work should be prioritized, ensuring adequate rest during the workday and days off from work. Today, it is current practice in Danish supermarkets to carry out the predominant part of stocking tasks during the early hours to ensure full shelves when opening the stores for customers. This entails an uneven workload between workers working morning shifts and workers working afternoon shifts, e.g., those workers working morning shifts performing the vast majority of the physically demanding stocking tasks. It could make sense to distribute stocking tasks, and hence, the workload, to more workers during the whole day instead of predominantly those working in the morning. Increasing variation in the physical demands could also be meaningful, and could include workers rotating between work tasks, job categories, or departments [14,18,20] with higher and lower physical demands and different exposure profiles, e.g., alternating heavy lifting, cashier work, and rest. Recently, a feasibility study has suggested that work can be re-organized and thereby reduce fatigue and pain while also increasing energy [59], and this could theoretically also work in supermarkets due to the high diversity in work tasks and hence variation in exposure profiles.

A previous study among supermarket workers indicated that use of a technical assistive device was suitable and associated with lowered physical workload [60]. One explanation for the lower workload could be that technical assistive devices allow adjustments of the lifting height. Knowing that low lifting heights are associated with increased low-back loading [35,38,54], increased and proper use of technical assistive devices could be worth pursuing in the supermarket sector. Having the right assistive devices for the work tasks at hand may be important as well, granted that differences in physical workload have been reported between different technical assistive devices [61,62]. In relation to this, a recent Danish study among young supermarket workers suggested accessibility and functionality of the technical devices as places for improvement worth focusing on in the occupational health and safety work in the supermarkets [63]. However, it should be noted that the overall positive evidence for the prevention of MSD by use of technical devices is not convincing, which could be due to unsuccessful implementation [21,22].

In addition, work environment professionals, working environment inspection authorities, and the individual supermarkets should be aware that the results of this and previous field studies [25] indicate that handling low loads in awkward positions or at high heights may also place large physical demands on the workers. Thus, both our data and previous reports [24,25] suggest that proper and work environment-friendly design of the supermarkets [16,64,65] may be relevant in terms of reducing the physical workload, for instance, by removing or adjusting the height of the lowest and highest shelves. In fact, some supermarket chains in Denmark have already integrated these work environment considerations into their physical store concepts. Future studies should investigate such developments in relation to the physical workload during stocking, as improving the store layout and shelf design could possibly alleviate peak and cumulative workloads during grocery stocking.

### 4.2. Strengths and Limitations

This study contains several strengths and limitations. Strengths include the comparably large sample size, the use of technical measurements instead of more bias-prone self-reports [31], diversity in the inclusion of different supermarket types and sizes increasing generalizability, and the within-subject repeated measures design increasing the statistical power.

Although our applied methodology aimed to minimize the risk hereof, EMG contains some general limitations such as difficulty establishing a valid maximum effort for sEMG normalization, signal dropout, cross-talk, and poor skin-electrode contact [66]. EMG collected during dynamic conditions can also be somewhat problematic, as the changing length and pennation angle of the muscle fibers can influence the amplitude and frequency content of the signals. Nevertheless, during high dynamic conditions with increasing workload, a consistent association between load and normalized EMG exists [67]. The fact that data were collected in the field during daily work is both a strength and weakness of the study. The real-world conditions increase the external validity but comes at the expense of lower internal validity granted that the lifts were not standardized. Considering that lifting is a dynamic movement, lifting factors other than lifting load and height influence the physical workload as well, e.g., asymmetry angle and horizontal location [38,68]. Thus, this study is an example of a simple biomechanical assessment compared to more advanced lifting indexes incorporating multiple lifting factors. We assessed peak muscular workload, but it still remains to be determined whether peak or cumulative loading is the strongest predictor of MSD [57,58]. It would yield a more realistic picture if we had measured during the whole workday and over several days instead of just one [69]. It is also reasonable to speculate that health status, i.e., occurrence of musculoskeletal pain, could have affected the participants’ work behavior, and we did not control for this. In addition, the EMG method is highly time-consuming and holds the risk of altered behavior due to the awareness of being observed (the Hawthorne effect).

## 5. Conclusions

These technical field measurements demonstrate a significant influence of load mass and lifting heights on muscular workload of the low-back and neck/shoulder muscles during grocery stocking across five supermarket chains. More specifically, a significant interaction was found between start and end position, e.g., lifts performed from ‘Low’ to ‘High’ were associated with a particularly high low-back muscular workload, whereas especially ‘High’ start and end positions demonstrated high workload of the neck/shoulders. These results can guide and should encourage preventive initiatives in the supermarket sector, such as work re-organization, re-designing shelf heights, and improved use of technical assistive devices.

## Figures and Tables

**Table 1:** Participant characteristics.

	*n*	Mean	SD	%
Age (years)	64	31.1	12.5	
Gender	64			
Women	25			39
Men	39			61
Height (cm)	56	175.3	10.8	
Weight (kg)	56	77.4	15.8	
Smoking	56			
Yes, daily	16			29
Yes, sometimes	5			9
Ex-smoker	10			18
No, never	25			45
General health	56			
Excellent	9			16
Quite good	19			34
Good	23			41
Not good	5			9

*n* = number, SD = standard deviation, % = percentage.

**Table 2:** Muscular workload by load mass. Estimates are presented as % nEMG (95% CI).

Load Mass (kg)	% nEMG	
	**Low-Back**	**Neck/Shoulders**
0–1	20 (18–21)	22 (20–23)
≥1–5	20 (18–22)	23 (22–24)
≥5–10	26 (24–28)	26 (25–28)
≥10–15	29 (27–31)	26 (24–29)
≥15	32 (30–34)	31 (29–32)

Muscular workload estimates color graded from green to red, with lower estimates marked as nuances of green and higher estimates marked as nuances of red. The analyses were mutually controlled for each predictive variable, participant age, sex, and muscle.

**Table 3:** Muscular workload by lifting start and end position. Estimates are presented as % nEMG (95% CI).

Lifting Height	% nEMG (95% CI)	
	**Low-Back**	**Neck/Shoulders**
Start-Low	27 (25–29)	23 (22–24)
Start-Mod	25 (23–27)	25 (23–26)
Start-High	24 (22–26)	29 (27–30)
End-Low	25 (24–27)	23 (21–24)
End-Mod	26 (24–28)	24 (23–26)
End-High	25 (23–26)	30 (29–31)

Muscular workload estimates color graded from green to red, with lower estimates marked as nuances of green and higher estimates marked as nuances of red. The analyses were mutually controlled for each predictive variable, participant age, sex, and muscle.

**Table 4:** Muscular workload of the lower back arranged by load mass and start and end position of the lift. The interaction between start and end position for muscular workload was statistically significant. Estimates are presented as % nEMG (95% CI).

Start	End	0–1 kg	≥1–5 kg	≥5–10 kg	≥10–15 kg	≥15 kg
Low	Low	20 (19–22)	20 (18–22)	30 (26–33)	34 (28–40)	33 (30–36)
Low	Mod	23 (21–25)	24 (22–26)	33 (29–36)	34 (27–40)	41 (38–44)
Low	High	25 (23–27)	26 (23–28)	34 (25–44)	58 (40–76)	46 (39–52)
Mod	Low	22 (20–23)	23 (20–25)	27 (24–30)	31 (25–37)	32 (30–35)
Mod	Mod	19 (17–20)	21 (19–23)	25 (22–28)	27 (20–33)	32 (29–35)
Mod	High	17 (15–19)	19 (17–21)	26 (22–31)	31 (12–50)	45 (37–52)
High	Low	20 (17–23)	27 (22–31)	30 (24–36)	34 (23–45)	35 (31–39)
High	Mod	18 (16–21)	19 (16–22)	27 (23–31)	28 (21–35)	34 (30–39)
High	High	15 (13–18)	18 (15–21)	26 (19–32)	N/A	N/A

Muscular workload estimates color graded from green to red, with lower estimates marked as nuances of green and higher estimates marked as nuances of red. The analyses were mutually controlled for each predictive variable, participant age, sex, and muscle. N/A = estimate not available due to low number of observations, i.e., low statistical power.

**Table 5:** Muscular workload of the neck/shoulders arranged by load mass and start and end position of the lift. The interaction between start and end position for muscular workload was statistically significant. Estimates are presented as % nEMG (95% CI).

Start	End	0–1 kg	≥1–5 kg	≥5–10 kg	≥10–15 kg	≥15 kg
Low	Low	17 (16–18)	18 (16–20)	18 (14–22)	16 (9–22)	22 (19–25)
Low	Mod	17 (16–18)	20 (18–23)	21 (17–26)	34 (27–41)	31 (28–34)
Low	High	23 (21–24)	28 (25–32)	34 (24–44)	28 (9–46)	44 (36–52)
Mod	Low	17 (16–19)	22 (20–24)	18 (14–22)	22 (16–28)	28 (25–30)
Mod	Mod	18 (16–19)	22 (20–24)	24 (20–28)	24 (18–30)	33 (30–36)
Mod	High	25 (23–26)	29 (27–31)	40 (35–45)	38 (19–58)	53 (44–63)
High	Low	23 (20–26)	30 (25–36)	30 (23–37)	38 (27–49)	42 (38–47)
High	Mod	23 (21–25)	27 (24–30)	32 (28–37)	27 (20–35)	45 (40–50)
High	High	23 (22–25)	27 (24–31)	34 (26–41)	N/A	N/A

Muscular workload estimates color graded from green to red, with lower estimates marked as nuances of green and higher estimates marked as nuances of red. The analyses were mutually controlled for each predictive variable, participant age, sex, and muscle. N/A = estimate not available due to low number of observations, i.e., low statistical power.

## Data Availability

The data presented in this study are available on request from the corresponding author. The data are not publicly available due to general data protection regulations (GDPR), and personal data ordinance (PDPO).

## Supplementary Materials

The following supporting information can be downloaded at: https://www.mdpi.com/article/10.3390/ijerph19053030/s1, Table S1. Differences in least-square means (DLSM) between conditions and *p*-value (*p*=); Table S2. Differences in least-square means (DLSM) between conditions and *p*-value (*p*=); Table S3. Differences in least-square means (DLSM) between conditions and *p*-value (*p*=); Table S4. Differences in least-square means (DLSM) between conditions and *p*-value (*p*=).
