# Supplementary material for: The Importance of Lifting Height and Load Mass for Muscular Workload during Supermarket Stocking: Cross-Sectional Field Study
Source: Int J Environ Res Public Health. 2022 Mar 4;19(5):3030. doi: 10.3390/ijerph19053030 (PMC8910655; doi:10.3390/ijerph19053030)
Supplement: Supplementary file 1 [file ijerph-19-03030-s001.zip › ijerph-1544837-supplementary.pdf]

Supplementary Tables S1-S4

Supplementary Table S1. Differences in least-square means (DLSM) between conditions and *p*-value (*p*=).

		Neck/shoulders		Low-back	
**Condition 1**	**Condition 2**	**DLSM**	***p*=**	**DLSM**	***p*=**
Start-High	Start-Low	5.64	<0.0001	−3.56	<0.0001
Start-High	Start-Mod	4.21	<0.0001	−1.26	0.0015
Start-Low	Start-Mod	−1.42	<0.0001	2.31	<0.0001
End-High	End-Low	7.34	<0.0001	−0.88	0.0028
End-High	End-Mod	5.64	<0.0001	−1.19	<.0001
End-Low	End-Mod	−1.70	<0.0001	−0.31	0.1489

Supplementary Table S2. Differences in least-square means (DLSM) between conditions and *p*-value (*p*=).

		Neck/shoulders		Low-back	
**Condition 1**	**Condition 2**	**DLSM**	***p*=**	**DLSM**	***p*=**
0–1 kg	>1–5 kg	−1.53	<0.0001	−0.24	0.3937
0–1 kg	>5–10 kg	−4.55	<0.0001	−6.03	<0.0001
0–1 kg	>10–15 kg	−4.96	<0.0001	−9.46	<0.0001
0–1 kg	>15 kg	−9.21	<0.0001	−12.25	<0.0001
>1–5 kg	>5–10 kg	−3.02	<0.0001	−5.79	<0.0001
>1–5 kg	>10–15 kg	−3.44	0.0002	−9.22	<0.0001
>1–5 kg	>15 kg	−7.69	<0.0001	−12.01	<0.0001
>5–10 kg	>10–15 kg	−0.41	0.6784	−3.43	<0.0001
>5–10 kg	>15 kg	−4.66	<0.0001	−6.22	<0.0001
>10–15 kg	>15 kg	−4.25	<0.0001	−2.79	0.0005

Supplementary Table S3. Differences in least-square means (DLSM) between conditions and *p*-value (*p*=).

Low-back											
		0–1 kg		>1–5 kg		>5–10 kg		>10–15 kg		>15 kg	
**Condition 1**	**Condition 2**	**DLSM**	***p*=**	**DLSM**	***p*=**	**DLSM**	***p*=**	**DLSM**	***p*=**	**DLSM**	***p*=**
Low-High	Low-Low	4.43	<0.0001	5.35	<0.0001	4.52	0.340	23.86	0.012	12.99	<0.0001
Low-High	Low-Mod	1.71	0.018	1.47	0.235	1.67	0.728	24.45	0.010	5.21	0.095
Low-High	Mod-High	7.68	<0.0001	6.42	<0.0001	7.94	0.106	26.90	0.041	1.28	0.790
Low-High	Mod-Low	3.35	<0.0001	3.02	0.016	7.43	0.115	26.85	0.004	13.40	<0.0001
Low-High	Mod-Mod	6.36	<0.0001	4.94	<0.0001	9.45	0.043	31.37	0.001	13.51	<0.0001
Low-Low	Low-Mod	−2.71	<0.0001	−3.88	<0.0001	−2.86	0.156	0.58	0.888	−7.78	<0.0001
Low-Low	Mod-High	3.25	<0.0001	1.06	0.188	3.41	0.148	3.04	0.752	−11.71	0.002
Low-Low	Mod-Low	−1.08	0.001	−2.33	0.003	2.91	0.103	2.98	0.422	0.41	0.592
Low-Low	Mod-Mod	1.93	<0.0001	−0.41	0.544	4.93	0.005	7.51	0.036	0.52	0.575
Low-Mod	Mod-High	5.96	<0.0001	4.95	<0.0001	6.27	0.009	2.46	0.806	−3.93	0.290
Low-Mod	Mod-Low	1.63	0.000	1.55	0.064	5.76	0.002	2.40	0.515	8.19	<0.0001
Low-Mod	Mod-Mod	4.65	<0.0001	3.47	<0.0001	7.78	<0.0001	6.93	0.061	8.30	<0.0001
Mod-High	Mod-Low	−4.33	<0.0001	−3.40	<0.0001	−0.50	0.819	−0.06	0.995	12.13	0.001
Mod-High	Mod-Mod	−1.32	0.001	−1.47	0.046	1.52	0.480	4.47	0.647	12.23	0.001
Mod-Low	Mod-Mod	3.01	<0.0001	1.92	0.006	2.02	0.195	4.53	0.179	0.10	0.913
High-Low	High-Mod	1.57	0.333	7.69	0.001	3.31	0.309	6.23	0.279	0.51	0.827
High-Low	Low-High	−4.88	0.001	1.30	0.586	−3.93	0.466	−24.08	0.020	−11.13	0.001
High-Low	Low-Low	−0.46	0.743	6.65	0.002	0.60	0.850	−0.22	0.971	1.86	0.244
High-Low	Low-Mod	−3.17	0.027	2.77	0.205	−2.26	0.485	0.36	0.952	−5.92	0.000
High-Low	Mod-High	2.79	0.049	7.72	0.001	4.01	0.236	2.82	0.795	−9.85	0.013
High-Low	Mod-Low	−1.54	0.272	4.32	0.047	3.51	0.250	2.76	0.625	2.28	0.157
High-Low	Mod-Mod	1.48	0.292	6.24	0.004	5.52	0.071	7.29	0.208	2.38	0.154
High-Mod	Low-High	−6.46	<0.0001	−6.39	<0.0001	−7.24	0.137	−30.32	0.002	−11.64	0.001
High-Mod	Low-Low	−2.03	0.024	−1.04	0.404	−2.71	0.218	−6.45	0.112	1.36	0.470
High-Mod	Low-Mod	−4.74	<0.0001	−4.92	0.000	−5.57	0.014	−5.87	0.183	−6.42	0.001
High-Mod	Mod-High	1.22	0.190	0.02	0.986	0.70	0.781	−3.41	0.729	−10.36	0.010
High-Mod	Mod-Low	−3.11	0.001	−3.38	0.007	0.19	0.925	−3.47	0.378	1.77	0.347
High-Mod	Mod-Mod	−0.10	0.914	−1.45	0.221	2.21	0.262	1.06	0.794	1.87	0.335
High-High	High-Low	−4.52	0.003	−8.61	0.000	−4.76	0.257	N/A	N/A	N/A	N/A
High-High	High-Mod	−2.95	0.005	−0.91	0.583	−1.45	0.679	N/A	N/A	N/A	N/A
High-High	Low-High	−9.40	<0.0001	−7.31	<0.0001	−8.69	0.112	N/A	N/A	N/A	N/A
High-High	Low-Low	−4.98	<0.0001	−1.95	0.141	−4.16	0.214	N/A	N/A	N/A	N/A
High-High	Low-Mod	−7.69	<0.0001	−5.84	<0.0001	−7.02	0.040	N/A	N/A	N/A	N/A
High-High	Mod-High	−1.72	0.013	−0.89	0.514	−0.75	0.835	N/A	N/A	N/A	N/A
High-High	Mod-Low	−6.06	<0.0001	−4.29	0.002	−1.25	0.703	N/A	N/A	N/A	N/A
High-High	Mod-Mod	−3.04	<0.0001	−2.36	0.069	0.76	0.812	N/A	N/A	N/A	N/A

Supplementary Table S4. Differences in least-square means (DLSM) between conditions and *p*-value (*p*=).

Neck/shoulders											
		0–1 kg		>1–5 kg		>5–10 kg		>10–15 kg		>15 kg	
**Condition 1**	**Condition 2**	**DLSM**	***p*=**	**DLSM**	***p*=**	**DLSM**	***p*=**	**DLSM**	***p*=**	**DLSM**	***p*=**
Low-High	Low-Low	5.83	<0.0001	10.46	<0.0001	16.34	0.0012	11.86	0.2247	22.08	<0.0001
Low-High	Low-Mod	5.81	<0.0001	8.10	<0.0001	13.12	0.0101	−6.10	0.5315	13.17	0.0008
Low-High	Mod-High	−1.81	0.0152	−0.76	0.6328	−5.51	0.2901	−10.85	0.4237	−9.45	0.1157
Low-High	Mod-Low	5.35	<0.0001	6.57	<0.0001	16.53	0.001	5.17	0.5884	16.42	<0.0001
Low-High	Mod-Mod	5.09	<0.0001	6.53	<0.0001	10.67	0.0316	3.70	0.6996	11.13	0.0047
Low-Low	Low-Mod	−0.01	0.9744	−2.35	0.0177	−3.22	0.1301	−17.96	<0.0001	−8.90	<0.0001
Low-Low	Mod-High	−7.64	<0.0001	−11.21	<0.0001	−21.85	<0.0001	−22.71	0.0237	−31.53	<0.0001
Low-Low	Mod-Low	−0.48	0.1701	−3.89	0.0001	0.19	0.919	−6.69	0.0773	−5.66	<0.0001
Low-Low	Mod-Mod	−0.74	0.029	−3.92	<0.0001	−5.67	0.0024	−8.16	0.025	−10.94	<0.0001
Low-Mod	Mod-High	−7.62	<0.0001	−8.86	<0.0001	−18.63	<0.0001	−4.75	0.6445	−22.63	<0.0001
Low-Mod	Mod-Low	−0.46	0.3317	−1.54	0.15	3.41	0.0789	11.26	0.0034	3.25	0.0021
Low-Mod	Mod-Mod	−0.73	0.1209	−1.57	0.0803	−2.46	0.1868	9.80	0.0094	−2.04	0.0938
Mod-High	Mod-Low	7.16	<0.0001	7.32	<0.0001	22.05	<0.0001	16.02	0.1125	25.87	<0.0001
Mod-High	Mod-Mod	6.90	<0.0001	7.29	<0.0001	16.18	<0.0001	14.55	0.1484	20.59	<0.0001
Mod-Low	Mod-Mod	−0.27	0.4532	−0.03	0.9711	−5.87	0.0004	−1.47	0.6684	−5.29	<0.0001
High-Low	High-Mod	−0.53	0.7557	3.42	0.2564	−2.14	0.5481	10.78	0.0706	−2.70	0.3475
High-Low	Low-High	−0.17	0.9167	2.05	0.5029	−4.09	0.4792	10.59	0.3196	−1.66	0.6947
High-Low	Low-Low	5.66	<0.0001	12.51	<0.0001	12.25	0.0005	22.45	0.0004	20.42	<0.0001
High-Low	Low-Mod	5.65	0.0001	10.15	0.0003	9.03	0.0109	4.49	0.4648	11.51	<0.0001
High-Low	Mod-High	−1.97	0.1807	1.30	0.6462	−9.60	0.0095	−0.26	0.9814	−11.12	0.0244
High-Low	Mod-Low	5.19	0.0004	8.62	0.0021	12.45	0.0002	15.76	0.0076	14.76	<0.0001
High-Low	Mod-Mod	4.92	0.0007	8.59	0.0019	6.58	0.0509	14.29	0.0176	9.47	<0.0001
High-Mod	Low-High	0.36	0.7487	−1.36	0.4948	−1.95	0.707	−0.19	0.9845	1.04	0.8159
High-Mod	Low-Low	6.19	<0.0001	9.09	<0.0001	14.39	<0.0001	11.67	0.0058	23.12	<0.0001
High-Mod	Low-Mod	6.18	<0.0001	6.74	<0.0001	11.17	<0.0001	−6.29	0.1618	14.21	<0.0001
High-Mod	Mod-High	−1.45	0.1382	−2.12	0.1849	−7.46	0.006	−11.04	0.2783	−8.41	0.0955
High-Mod	Mod-Low	5.71	<0.0001	5.20	0.001	14.58	<0.0001	4.98	0.2147	17.46	<0.0001
High-Mod	Mod-Mod	5.45	<0.0001	5.17	0.0005	8.72	<0.0001	3.51	0.3915	12.17	<0.0001
High-High	High-Low	0.77	0.6242	−3.10	0.3227	3.65	0.4233	N/A	N/A	N/A	N/A
High-High	High-Mod	0.24	0.8279	0.31	0.8812	1.51	0.6866	N/A	N/A	N/A	N/A
High-High	Low-High	0.60	0.5106	−1.05	0.6189	−0.44	0.9402	N/A	N/A	N/A	N/A
High-High	Low-Low	6.43	<0.0001	9.41	<0.0001	15.90	<0.0001	N/A	N/A	N/A	N/A
High-High	Low-Mod	6.42	<0.0001	7.05	<0.0001	12.69	0.0005	N/A	N/A	N/A	N/A
High-High	Mod-High	−1.21	0.0944	−1.80	0.3013	−5.95	0.122	N/A	N/A	N/A	N/A
High-High	Mod-Low	5.95	<0.0001	5.52	0.0017	16.10	<0.0001	N/A	N/A	N/A	N/A
High-High	Mod-Mod	5.69	<0.0001	5.48	0.001	10.23	0.0029	N/A	N/A	N/A	N/A
